# Infantile Paroxysmal Convulsions, and Their Treatment with Sulphate of Quinine, with Cases

**Published:** 1849-11

**Authors:** Henry F. Campbell

**Affiliations:** Demonstrator of Anatomy in the Medical College of Georgia


### Part 3.—Selections.

ARTICLE I.

*Infantile Paroxysmal Convulsions, and their Treatment with Sulphate of Quinine, with Cases.*—By HENRY F. CAMPBELL, M.D., Demonstrator of Anatomy in the Medical College of Georgia.

* * * * * From an attentive observation of many cases which our locality so abundantly affords, we are disposed to an opinion, somewhat varying with that inculcated by most reports concerning the *time* of the occurrence of the infantile convulsions of intermittent fever, viz:—that they more frequently than otherwise, if not invariably, occur at the beginning of the paroxysm, or during the chill, and not at the acme of fever; and consequently, they are not the result of a high degree of vascular excitement as has been, I believe, generally supposed. Now, it is common to find them coming on at a time when there seems to be least disturbance in the nervous or vascular system; for instance, a child will be playing or running about apparently well, when suddenly it is attacked with a violent convulsion, often with others succeeding, and after their subsidence, the case will be found to assume all the features of an ordinary intermittent paroxysm, progressing regularly on to intermission, &c. Indeed the convulsion seems to take the place of the cold stage, and is, so to speak, a chill very much exaggerated, that is the normal innervation which in a subject less favorable, would have produced only a *chill*, here in the extremely mobile and impressible nervous system of a child, gives rise to phenomena of graver and more alarming character, and the paroxysm is *ushered in* by a *convulsion.* We would here advert to an example which may be considered a transition case between the chill and the convulsion; and developes, to a certain extent, our view regarding the character of the convulsion, and the relation it bears to the paroxysm. E. P., a young woman of nervous temperament and general bad health, aged about 22 years, on the advent of her second paroxysm, was affected with involuntary contractions in the muscles of the arms and legs. I would not be misapprehended, these were not the ordinary quaking and subsultus of an ague, but of such a marked character as to assume decidedly the form of a convulsion; but not to the extent however of the deprivation of consciousness. She was fully aware when they were about to commence, and would call to her attendants for assistance in preventing their accession; here the ordinary ague-shaking was evidently exaggerated into a true convulsion. On examination by pressure of the spine, we found the dorsal and lumbar regions extremely sensible. On the removal of sinapisms from the legs (where they had been very irrationally applied) to the spine, the convulsions were relieved in a short time, and the administration of Quinine, during the intermission, prevented the return of the paroxysm. As the spinal irritation was of long standing, it was deemed expedient in this case, to apply a blister, a more permanent revulsive.

Thus fully impressed with the analogy between the cold stage and the convulsion, and having, in a few instances, succeeded in arresting the progress of a paroxysm, by the administration of Quinine, even after the commencement of the chill, and finding also, that when it did not entirely succeed in arresting the paroxysm, it generally mitigated its violence, thus disproving the gratuitous and pernicious dogma, that "where Quinine does not cure, it makes worse," we have been induced to use this remedy empirically in several obstinate cases of infantile convulsions of this character. While we report them, with some degree of confidence, as suggestive *experiments*, worthy, perhaps, of further investigation, yet we will not admit for the practice, any thing like established merit, as the cases are too few to deserve more than the credit perchance of exciting *enquiry* into the value of the application.

In the relation of the following cases we have not deemed it necessary, to recount minutely the history of each, as they are, in most respects, but ordinary cases of intermittent fever, and the history of one of them is but the prototype of the others.

Case 1. H. H., a boy, aged 2 years, had had a paroxysm of intermittent fever, previously, while playing about on the floor, was seized with a convulsion, which was quickly succeeded by others, which resisting the ordinary means used for their relief, we resorted to the administration of Quinine, grs. 2½ every hour till grs. 7½ were taken. The convulsions subsided after the second dose. The calomel, previously given, acted upon the bowels, and the return of the paroxysm was prevented by Quinine given in anticipation next day.

Case 2. Edward Bleese, aged about 6 years, had been the subject of tertian intermittent fever for a week. We were called to see him in a convulsion which came on at the beginning of the paroxysm. When seen, he had had many convulsions. The bowels were emptied by oil and enemata—mustard plasters were applied to the spine, abdomen and extremities, without exerting any controlling influence on the convulsions. The convulsive action was almost incessant.—Ten grains of Quinine were administered at a single dose; in about an hour after, the convulsions became less frequent and finally entirely subsided. The succeeding paroxysm was met with Quinine, and the patient was afterwards treated for worms with very abundant results.

Case 3. A. McGraef, a boy of very delicate and unhealthy appearance, aged about 12 years, was seized with a convulsion on the accession of his third or fourth paroxysm. He had had no treatment previous to this call. Dr. J. L. Watkins, then our pupil, attended the case, and having applied the usual routine of remedies, as sinapism, enemata, pediluvia, &c., the following dose was administered: Quinine grs. 10; Calomel grs. 15. The mustard plasters, pediluvia, &c., were continued with the application of cold to the head, and 5 grains of Quinine were given in an hour after the first dose. The convulsions ceased in about half an hour after the administration of the medicine. The sweating stage quickly succeeded, and on the next day the boy was apparently as well as ever. Of course more than the ordinary caution was observed to prevent another paroxysm, by Quinine the next day.

We also constantly remark, that these convulsions are found to attend the hebdomadal relapses of the paroxysmal fever, upon which they depend, as the following will show:

Case 4. S., a child, aged 2½ years, of excellent general health and ruddy appearance, was taken at the first paroxysm with a convulsion, while apparently quite well. When we saw this case, the convulsions had continued for more than an hour with but short intermissions. After ascertaining that the child had not lately taken any crude ingesta that required removal, we administered of Hyd. Chlor. Mit. 6 grains; Quinine 8 grains, in one powder. The convulsions ceased in about an hour after this dose, and on the operation of the calomel, the fever subsided and the case regularly proceeded through phases of an ordinary infantile intermittent, and was subsequently treated accordingly. In about three weeks after, a relapse occurred attended with convulsions. The Quinine was given in a single dose of 5 grains in combination with calomel. The douche to the head and sinapisms to the spine were also applied. The Quinine was here given earlier, and the case was of shorter duration than before.

We could here advert to other cases, one occurring under the observation of our friend Dr. L. D. Ford, of this city, wherein the hebdomadal return of the paroxysm is invariably attended with a convulsion in its approach, and another similar one, in our own practice, wherein the administration of minute doses of Fowler's solution in the interval prevented the paroxysm and the attending convulsion, but our limits will not admit of their full report.

Above we have given a faithful, though not a minute recountal of four cases of convulsions, treated with the sulphate of Quinine during the attack. We are free to admit with due candor, that they are far from conclusive; their paucity, as well as the mixed treatment to which such cases must necessarily be subjected, detract much from their weight, and render us cautious in our opinion of their results. We are aware that our opinion, the opinion of medical men generally, respecting the effect of their remedies, is apt to be influenced by prejudice, and determined by *á priori* conclusions, and should be treated by the profession with some degree of distrust, but still, as we have before remarked, we would attach some little importance to the results of our observations on this mode of practice, and although we cannot recommend Quinine as an only remedy to be depended on in the treatment of this class of infantile convulsions, we cherish the hope that this report, imperfect as it is, may instigate further investigation on this subject, so important to the profession and to the world.—*Southern Med. and Surg. Journal.*

